# The Teeth and Their Preservation

**Published:** 1852-01

**Authors:** 


*The Teeth and their Preservation.* By Charles Vasey, Dentist, Renshaw, London.

The author having found that the teeth of many persons are in a measure neglected and destroyed, for the want of a little knowledge, was induced to publish the little work before us in a popular form.

The book is exceedingly well written, and conveys to the general reader, sound and judicious information upon the teeth.

